# Role of the Neurons, Astrocytes and Particle-Wave Duality in Conventional Electromagnetic Field, Plasma Brain Dynamics and Quantum Brain Dynamics

**DOI:** 10.21315/mjms2021.28.6.1

**Published:** 2021-12-22

**Authors:** Zamzuri IDRIS, Zaitun ZAKARIA, Jafri Malin ABDULLAH

**Affiliations:** 1 Department of Neurosciences, School of Medical Sciences, Universiti Sains Malaysia, Kubang Kerian, Kelantan, Malaysia; 2 Brain and Behaviour Cluster, School of Medical Sciences, Universiti Sains Malaysia, Kubang Kerian, Kelantan, Malaysia; 3 Hospital Universiti Sains Malaysia, Universiti Sains Malaysia, Kubang Kerian, Kelantan, Malaysia

**Keywords:** astrocytes, cognitions, consciousness, electricity, neural binding, plasma brain dynamics, psychosis, quantum brain dynamics

## Abstract

The brain is regarded as the most complex anatomical structure in the human body that executes various functions. The electromagnetic brainwaves or energy with its discrete network is commonly thought of as the sole contributor to various brain functions. However, the discrete pattern of the brain network seems insufficient to explain consciousness, binding problems in neural communication, brain heat, psychiatric manifestations and higher-order of thinking. Therefore, it seems that the brain must possess additional energy for it to have a much higher degree of functional freedom. Plasma brain dynamics (PBD) and quantum brain dynamics (QBD) are two hypothetical brain energies that have a strong scientific basis to exist together with the conventional brain electromagnetic energy. The presence of these energies may explain the puzzling brain functions, thus creating an opportunity to correct any abnormalities arising from them.

## Conventional Electromagnetic Field, Light-Quantum Field and Plasma Brain

Neural communication is known to be related to electricity and magnetic fields. The flow of electromagnetic fields has been linked to a particular neuroanatomical network for a specific function, known as the brain network. This concept views the brain as a Newtonian or classical brain, with an ensemble of particles forming the anatomy, and from it arises the physiological functions. This concept alone however could not explain several functions related to the brain, such as: i) consciousness; ii) binding problem; iii) cognitions with a high degree of freedom such as creativity or abstract thinking and iv) psychiatric manifestations.

**Figure 1:**
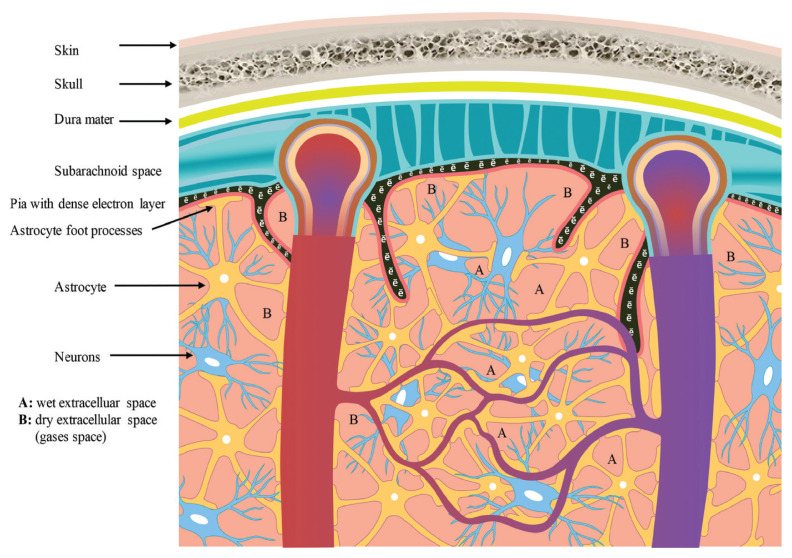
The wet and dry extracellular spaces. (A) The wet extracellular space has more fluid but is limited inside the brain. On the contrary, the dry extracellular space, (B) has a significantly lesser fluid volume, containing more gases, and is abundant inside the brain. The astrocytes with their Aquaporin-4 create the relatively dry extracellular spaces, which are thought of as important in generating brain plasma vortex energy. Regarding the neuron, it is a conventional view that it propagates the neural action potential, and with a group of neurons, electromagnetic brainwaves are generated. The third energy is quantum energy, whereby anatomy itself can be viewed as energy simply because the atom (brain anatomy) can exist as either ensemble of particles or purely waves form of energy. Any defect in the brain’s anatomy could alter the brain’s energy

In this editorial, the authors invite the reader to consider an additional concept for brain function — the quantum plasma brain. It is a fusion of quantum brain dynamics (QBD) with plasma brain dynamics (PBD) (1, 2) and the essence of this concept is light and ionised gases. The light comprises dual features, either particle (photons) or waves, and the authors described it as the gist for the light-quantum field (light-QF) in the human brain, which is low dimensional, diffuse, and interconnecting with the cosmos quantum field, widespread, non-directional, changing, unlocatable, high entropy and symmetry (coherence) (3, 4). The QBD, PBD and the conventional brain electromagnetic field (EMF) form the total brain field (mind or fused field) for neural communication. Another interesting fact is revisited in this article regarding ionised gases or plasmas. In 2009, Nakada (5) published a review on the neuroscience of water molecules. Besides conventional fluid-filled brain extracellular space (wet area), which is small in the brain when compared to other organs, the researcher also highlighted the importance of astrocytes Aquaporin-4 (also abundant in renal tubules) in creating dry or gas-filled extracellular spaces (dry area) inside the brain. The dry area does not mean it has no fluid at all, instead, the amount is much reduced when compared to the brain wet area and the important or abundant gas in it is thought of as carbon dioxide (CO_2_) (Figure 1).

A high concentration of carbonic anhydrase in oligodendroglia is one of the reasons to believe that CO_2_ would be the primary gas within the dry area. Interestingly, CO_2_ is easily converted to ionised gas or plasmas when electricity is applied to them even in mild conditions. The electricity or electron movements that trigger this conversion are likely coming from the conventional EMF. The higher the EMF energy (more communication of information), the higher plasma conversion occurs. It has been established that plasmas are very good energy conductors (vortex/flow of energy) and they can generate and renew electricity (6). Another important point is the surrounding temperature of the plasma conversion area that also determines the completeness of the conversion — some are partially ionized while others are fully ionised gases (plasmas).

The generation of energy vortex through ionised gases is thought to occur in vast areas of the brain, especially at the cortical mantle, brain nuclei and brainstem. The adult human brain with an average weight of 1,500 g has 86 billion neurons; 1,000 trillion synaptic connections and 85 billion non-neuronal cells (7). Therefore, one may perceive that the matrix of the astrocytes covers vast areas of the brain and the generation of energy vortex in the dry area is believed to be related to the third type of neural communication or energy transfer (PBD) which can also generate heat. Thus, one may notice the propagation of wave vortices (with heat) from the deep (brainstem area) to the superficial cortices. This energy movement (vortex) is likely parallel with ascending energy of the EMF, which involves the thalamus and the hypothalamus (ventral and dorsal pathways for the electromagnetic brainwaves) (8).

The presence of an electron-dense layer at the pia or leptomeningeal layer (brain cortical surface just above the astrocytes foot processes) is another interesting fact about astrocytes. This feature is conceived to be associated with the spread of plasma energy vortex on the brain surface and integration of upcoming energy vortex from dry areas of the brain (PBD) through the cortical column system, which has been previously established by the conventional cortical neuroanatomy and the EMF (9). The presence of conventional EMF, diffuse light-QF (even interacting with the cosmos QF) or QBD, and ionised gases or plasma vortex energy (generate energy vortex and heat) or PBD makes the brain possess both a discrete EMF network (conventional EMF) and a high degree of functional freedom, which is very fast and capable in interacting meaningfully with the cosmos (QBD and PBD).

## Interaction of Light and Electricity and Bose-Einstein Brain

Based on the aforementioned discussion, the authors concluded that three types of energy are present in the human brain: i) electric (magnetic field) energy; ii) light energy (light-QF: based on an atom existing either in particles or waves form, or by describing anatomy itself as energy) and iii) plasma (ionised gases) energy vortex (with heat). It is noteworthy that at quantum or nanoscale, the interaction of light with electricity can also create plasmas. This unthinkable phenomenon has been demonstrated by using nanophotonics and nanoplasmonics in combination with magnetism (nanoscale magnetophotonics) (10). Whenever a magnet is pointed at a metal object, movement will occur due to the existence of a magnetic field. However, nothing seems to happen when the magnet is pointed to the light. The reason light and magnetism do not recognise one another is because of the much higher oscillation of light photons than the magnetic field oscillations. This was different when the electrons were exposed to light, there was an interaction that produces nanoplasmonic. This is attributed to the fact that electron oscillation is almost as fast as the light photon. With this observation, the authors suggest that there is a possibility that the first two brain energies: EMF (more of electric field/electrons) can interact with the light-QF and hence produce the brain plasmas at the nanoscale level. Thus, brain plasmas seem to be generated in two ways — the interaction of the light with electrons (electricity) and astrocytes dry area (filled with gases) triggered by electric pulses or heat. At this point, one may notice the presence of a conventional Newtonian view of the brain with its anatomy and physiological brainwaves at macro-, meso- and microscopic levels. At a deeper or quantum realm or nanoscopic level (100 nm or less, 1 nm = 10^−9^), quantum perspective is counted for. At this quantum level, the sciences of quantum mechanics are considered. Light-QF results from our quantum mechanics perspective of the brain. As stated before, the third type of brain energy is ionised or plasma vortex energy with its heat (thermal) production. Therefore, one may say the brain has both partially mechanical (classical and quantum mechanics) and partially thermodynamical systems.

At a macroscopic level, the brain temperature is tightly regulated at 36.9 °C ± 0.5°C–1.5°C (11–13). The regulatory mechanism could in fact involve this plasma energy vortex with cerebrospinal fluid bath, blood supply, air at the skull base and in the air sinuses, and black box irradiation inside the skull (heat regulation involving convection, conduction, radiation and evaporation). On the contrary, at the quantum or nanoscale level, its temperature may be different. Our thought is that, at this junction, near-zero Kelvin may play a role because at the Bose-Einstein condensate level, there is a persistent presence of wave coherence (at that temperature, the atoms all drop into the same quantum state: coherence) that drives the brain function from bottom to top hierarchy (14–15). In other words, wave coherence plays a pivotal role for brainwave parameters to be identified, studied and monitored. It may signify the healthy state of an individual (16–17). In summary, one may view the brain function according to: i) conventional Newtonian view — its brain anatomy and physiological waves; or ii) quantum view — with quantum physics principles applied to the view and lastly iii) plasma energy-vortex view together with its heat — the thermodynamics view of the brain. The interaction amongst these three brain energies may explain the unexplainable brain functions.

## The Three Brain Energies Explaining the Puzzling Brain Functions

These three types of brain energy explain: i) the typical brain network patterns for a specific function (conventional EMF) and ii) the unexplainable brain functions (involving the QBD and PBD or quantum plasma dynamics [QPD]: fusion of these two). Consciousness is one of the unexplainable functions. It is fundamental and seems to cover not only the brain but also the energy of the cosmos (18–20). This notion seems suited for the light-QF (QBD), which covers our brain and also the cosmos. A detailed explanation of this aspect was published by us in early 2021 (3). The binding problem in neural communication is another challenging topic, whereby a person’s brain binds all the pieces of brain input, such as seeing a thin guy wearing a blue jacket running towards you. The brain must bind all the pieces — a thin guy, blue colour and motion. The binding process has so far not been associated with any brain network. Thus, we suggest that this role may be executed either by the QBD or PBD or a combination of both. The coherence waves may bind all these pieces in a relatively fast manner. Thus, one may also say, the presence of QBD and/or PBD may explain the very fast function of the brain for distant communication, and the amount of wave coherence is important in health and disease. Incoherent waves may make the binding process incomplete, thus eliciting various split phenomena as observed in many psychiatric disorders. Regarding an accumulated heat or regulation of brain thermodynamics in PBD, head injury clinical studies have shown better clinical outcomes in those receiving direct cooling therapies (12–13). This signifies obtaining energy and heat homeostasis is vital in a severely injured brain. Given the aforementioned explanation, these events are most likely involving the PBD. Finally, one may observe that the brain has a small magnetic field which measured in the range of femto- to pico-tesla using the magnetoencephalography and has a small voltage or current relative to the external voltages or currents. The neurosurgeon will not get an electrical shock when they are operating in the rich neuronal areas, such as the brainstem. Hence, some sort of energy deduction or harmony has occurred inside the brain. This capability is thought of as involving a complex mechanism, not simply via a conventional EMF, but also integration of all these three energies.

## Conclusion

The brain is an anatomical structure that has complex underlying mechanisms. This complexity is important for it to have a high degree of functional freedom. Therefore, the presence of a discrete EMF network alone is insufficient to explain total brain functions. The unexplainable brain functions may involve additional energy fields or vortex (flow of energy): the light-QF (QBD) and the plasma vortex of energy (PBD). Based on these arguments, neurons, astrocytes (other supporting cells) and particle-wave duality concept in quantum physics is thought to contribute significantly in generating these three brain energies. Table 1 summarises the content.

## Figures and Tables

**Table 1:** The three brain energies and their features. Interaction amongst them likely exists

Brain energy	Conventional brain energy (EMF)	Plasma brain (PBD)	Quantum brain (Light-QF or QBD)
Perspective	Classical or Newtonian	Ionic gases with the heat energy vortex	Quantum mechanics perspective
System	Classical mechanical system	Thermodynamical system	Quantum mechanical system
The structure	Mainly neuron	Mainly astrocytes, other supporting cells and dry extracellular space	Quantum level of all anatomy: neurons, supporting cells, microtubules, spaces, etc.
Brain network	It is currently a fact for brain functions, especially those related to sensory, motor, auditory and vision with their networks	The network concept is vast and may start from the centre or deep inside the brainstem-thalamus/hypothalamus, and end at the surface of the cortices. Thus, it may involve neural binding, heat regulation and other brain functions	The network concept is unclear but it may fuse with EMF (light + electric at the nanoscale level: producing more plasmas) and may interact with the cosmos quantum field. Thus, it involves consciousness, cognition and psyche
Coherence	Minor role	Major role	Major role
Degree of functional freedom	Minimal because it is discrete and limited to a specific network	Moderate with gaseous plasmas covering whole-brain matrix	Maximal, it can also involve the cosmos energy
Heat generation	Moderate (specific path)	Maximum (whole brain and specific path)	Minimum (dispersed/widespread)
How to modify it?	Give diffuse or focal electric or magnetic field (physiology) (such as deep brain stimulation and transcranial magnetic stimulation) Correct the anatomy	Through heating or cooling at the surface or core of the brain Through non-invasive or surgical modification at the leptomeningeal surface (direct brain cooling or cisternostomy) Correct the anatomy	Light therapy such as deep brain light stimulation (Doppler broadened lineshape for the nucleus), transcranial or intracranial (areas/lobes), trans-nasal (inferior brain areas) or trans-oral (brainstem) light therapy. Finally, a room with light therapy Correct the anatomy
